# Draft Genome Sequence of *Pseudomonas fluorescens* LMG 5329, a White Line-Inducing Principle-Producing Bioindicator for the Mushroom Pathogen *Pseudomonas tolaasii*

**DOI:** 10.1128/genomeA.00383-13

**Published:** 2013-07-25

**Authors:** Maarten G. K. Ghequire, Hassan Rokni-Zadeh, Peyman Zarrineh, René De Mot

**Affiliations:** Centre of Microbial and Plant Genetics, University of Leuven, Heverlee-Leuven, Belgium

## Abstract

*Pseudomonas tolaasii*, the causative agent of *Agaricus bisporus* brown blotch disease, can be identified by the white line reaction, occurring upon confrontation of the tolaasin-producing mushroom pathogen with “*Pseudomonas reactans*,” producing the lipopeptide white line-inducing principle (WLIP). The draft genome sequence of the WLIP-producing indicator *Pseudomonas fluorescens* strain LMG 5329 is reported here.

## GENOME ANNOUNCEMENT

The white line test (1) enables the detection of the mushroom pathogen *Pseudomonas tolaasii*, relying on formation of a visible precipitate in the agar medium between colonies of this pathogen and an indicator strain, referred to as “*Pseudomonas reactans*.” This diagnostic phenotype stems from the interaction of the *P. tolaasii* virulence factor, tolaasin, with another diffusible cyclic lipopeptide, the white line-inducing principle (WLIP) from *P*. *reactans* (2). The WLIP biosynthetic system of the white line-indicator organism *Pseudomonas fluorescens* LMG 5329 (NCPPB 3149) was characterized (3). Here, we report the draft genome sequence of this mushroom isolate that is closely related to *P. fluorescens* SBW25 (4) and other *P. fluorescens* subclade 3 strains (5).

Genomic DNA was subjected to 100-cycle paired-end sequencing with an Illumina HiSeq 2000 (Genomics Core Facility, KU Leuven). Following FastQC quality assessment of raw data (http://www.bioinformatics.babraham.ac.uk/projects/fastqc), reads were trimmed to 50 bp for *de novo* assembly by Velvet (6). Assembly of 16,486,578 reads (56-fold median coverage) yielded 293 contigs with an N_50_ value of 55,400 bp. The total assembled length is 6,875,357 bp, with a G+C content of 60.5%, and the largest contig is 164,530 bp. Automated annotation using RAST (7) assigned a total of 6,324 protein-coding genes, along with 63 tRNAs.

In addition to WLIP nonribosomal peptide synthetase (NRPS) genes, the LMG 5329 genome carries a *pvfC* homologue, which is involved in the production of a putative signaling molecule (8), and four pyoverdine NRPS genes, suggesting the production of a decapeptidic siderophore identical to *P. fluorescens* strain 18.1 pyoverdine (9) but different from the heptapeptidic *P. fluorescens* SBW25 pyoverdine (3, 10). In addition, systems for the acquisition of Fe^2+^ (11) and heme (12) are present. The importance of iron metabolism for the organism is further inferred from multiple extracytoplasmic function (ECF) sigma factors assigned to the regulation of iron metabolism. The ECF complement includes a component of the cell surface-signaling system PUMA3 (13). Several genes can be linked to surface-associated determinants of a sessile lifestyle: genes encoding poly-β-1,6-*N*-acetylglucosamine (14), alginate, Pel and Psl polysaccharides (15), Fap amyloid protein (16), Flp pili (17), and phosphate-binding protein PstS (18). The proteome includes no homologues of the cell surface proteins LapA or LapF (18). In addition to the Tat secretory genes, clusters for type I, type II, type III (two systems), type V, and type VI secretion and for fimbrial chaperone-usher systems are predicted. Interbacterial antagonistic proteins include a nuclease-type soluble (S) pyocin, Rhs proteins, and a contact-dependent inhibitory factor (19, 20). Also notable is the apparent acquisition of a mobile element encoding mercury reductase-based resistance as found in *Pseudomonas putida* (21) and a β-proteobacterial-type methylamine utilization operon (22).

### Nucleotide sequence accession numbers.

This whole-genome shotgun project has been deposited at DDBJ/EMBL/GenBank under the accession no. ASGY00000000. The version described in this paper is version ASGY00000000.1.
